# Acceptance Factors and Barriers to the Implementation of Digital Interventions in Older People with Dementia and/or Their Caregivers: An Umbrella Review

**DOI:** 10.3390/jcm14227974

**Published:** 2025-11-10

**Authors:** Ricardo Madeira, Dulce Esteves, Nuno Pinto, Alessandro Vercelli, Maria Vaz Pato

**Affiliations:** 1 Department of Sport Sciences, University of Beira Interior, 6201-001 Covilhã, Portugal; 2 Research Centre in Sport Sciences, Health Sciences and Human Development (CIDESD), 6201-001 Covilhã, Portugal; 3 RISE-Health-UBI, University of Beira Interior, 6201-506 Covilhã, Portugal; 4 Faculty of health Sciences, University of Beira Interior, 6201-506 Covilhã, Portugal; 5 Department of Neuroscience Rita Levi Montalcini, Neuroscience Institute Cavalieri Ottolenghi, 10043 Turin, Italy

**Keywords:** barriers, acceptance, telemedicine, mobile applications, cognition, aging

## Abstract

**Background/Objectives:** Digital interventions are essential for dementia care, particularly for older and isolated populations, and provide valuable support for caregivers. This umbrella review aimed to evaluate the acceptability and barriers to implementing the use of digital tools for health monitoring and management in older people with dementia and/or their caregivers. **Methods:** The review included studies assessing acceptability factors and barriers related to technology use in these groups. A total of 612 studies were identified across three databases. After removing duplicates, 400 articles remained. Following title and abstract screening, thirty articles were selected for full-text evaluation and five met the eligibility criteria for inclusion in this review. These systematic reviews collectively covered 93 primary studies, encompassing 12 to 279 participants with dementia and 11 to 2761 caregivers. **Results:** Frequently reported factors included self-management support, information access, and enhanced communication, although these were not consistently addressed across all reviews. The most significant barrier was a lack of technical knowledge, which hindered effective use. This gap in knowledge could compromise self-management and potentially increase burden on caregivers. **Conclusions:** In conclusion, digital interventions offer significant benefits in addressing accessibility challenges and are generally well-received by people with dementia, their caregivers, and healthcare providers. However, addressing the lack of technological proficiency is crucial to ensuring these interventions are effective and do not inadvertently create additional challenges. Practical strategies should include tailored digital literacy training for older adults and caregivers, simplified user interfaces, and ongoing technical support to enhance engagement and long-term adherence.

## 1. Introduction

With advances in medicine, the increase in average human life expectancy has given rise to numerous health problems [1]. Most elderly people have declines in physical performance and cognitive abilities and sensory changes, which are associated with a high risk of falls, increased memory loss, and difficulty in performing autonomous daily tasks such as eating, dressing, administering medication, and shopping [1]. Deterioration in cognition has an impact on occupational, domestic, and social functioning [2], making it difficult to communicate and manage activities of daily living [3,4]. Dementia is common in old age and is associated with a significant decline in cognitive function, resulting in interference with autonomy, reduced physical function, and memory loss, and affecting the performance of daily tasks and socialization [1,2,3,4].

It is estimated that the number of people with dementia (PWD) will be 131.5 million by 2050 [5,6,7], with an economic cost of more than a trillion dollars a year [8,9]. According to the World Health Organization (WHO), more than 55 million people currently live with dementia worldwide, and almost 10 million new cases are diagnosed each year [10]. Dementia is the seventh leading cause of death in the world [10], thus arousing the interest of the scientific community and the WHO, and many policies to improve dementia [11] are being developed worldwide.

The lack of autonomy and security of PWD raises problems that affect not only patients but also carers [12,13,14]. The complexity of this disease has a physical, psychological, social, and economic impact on people with dementia, carers, families, and society in general [13,15,16,17]. This impacts on the lives of carers of people with dementia, resulting in frustration, stress, lower quality of life (QoL), health problems, and less time to look after themselves [12,15,17,18,19,20,21], producing a decrease in care quality for the patient.

The COVID-19 pandemic has highlighted the importance of innovative technologies to help healthcare professionals, caregivers, and frail old people. Technology and healthcare have emerged as allies in the fight against pathological aging [7,22,23]. Technology is a potential instrument for providing new means of monitoring, interactive communication (telehealth), and self-management of health [11,24]. However, the use of technology to assist and monitor people with dementia and their carers should be carefully evaluated, as some studies report that older adults may encounter barriers to adoption or express concerns about technological change. At the same time, other research highlights that many older adults can and do engage positively with technology when it is accessible, relevant, and user-friendly [22]. It is important to understand how technology can promote the autonomy and safety of people with dementia and their carers, as well as factors relating to its acceptability and the barriers to its use.

Understanding technology acceptance among older adults with cognitive impairments is inherently complex. Classical frameworks such as the Technology Acceptance Model (TAM) [25] and the Unified Theory of Acceptance and Use of Technology (UTAUT) [26] highlight perceived ease of use and perceived usefulness as key determinants of technology adoption. However, when applied to populations with cognitive decline, these models require specific adaptations, as factors such as memory, attention, and digital self-efficacy can strongly influence perception and engagement with technology. Recent research also indicates that emotional and contextual elements—such as caregiver support, trust in digital interfaces, and a sense of autonomy—play critical roles in shaping technology acceptance [27,28,29,30]. Therefore, a comprehensive theoretical framework should integrate these cognitive, emotional, and contextual dimensions to better capture the barriers and motivations underlying technology use among older adults with dementia.

To the best of our knowledge, this is the first umbrella review to focus explicitly on the acceptability of, and barriers to, the use of digital technologies by individuals with dementia and their carers. Although several systematic reviews and meta-analyses have examined related aspects, their findings remain fragmented and, at times, contradictory. Some reviews have focused narrowly on specific types of technologies or populations, while others have adopted heterogeneous methodologies, making it difficult to draw generalizable conclusions. Moreover, variations in methodological quality, publication bias, and inconsistent reporting of outcomes further limit the reliability and comparability of existing evidence.

By synthesizing and critically evaluating this body of research, the present umbrella review aims to provide a comprehensive and up-to-date overview of current knowledge, identify consistent patterns and gaps, and clarify the most robust evidence regarding acceptance factors and barriers to digital interventions among people with dementia and their carers. In doing so, it seeks to contribute to the theoretical debate and offer guidance for future research and policymaking. Ultimately, by categorizing both the barriers and facilitators related to technology use, this review aims to support the identification of technologies that have proven effective, as well as the challenges that hinder their broader implementation in promoting active ageing and supporting carers.

Several research questions were addressed:

1. What are the barriers to implementing digital interventions for people with dementia and/or their caregivers?
2. What are the facilitators to implementing digital interventions for people with dementia and/or their caregivers?
3. What technologies have been proposed for people with dementia and/or their caregivers?
4. How effective were these digital interventions in alleviating the targeted problems?

## 2. Methods

### 2.1. Literature Search

This umbrella review is registered on PROSPERO (registry number CRD42023409205). The protocol for this umbrella was accepted for publication (https://doi.org/10.2196/56584).

The ISI Web of science, Scopus, and Medline/PubMed databases were used to search for systematic reviews for the development of this umbrella. This review included studies on acceptability factors and barriers to implementing digital interventions for people with dementia and/or their caregivers. In this search, articles were identified using keywords and Mesh words (for PubMed searches), individually and/or in combination (see Supplementary File S1). We used a PICOS framework (population, intervention, comparison, outcomes, and study design (Table 1)). To implement this umbrella review, we used the Preferred Reporting Items for Overviews of Reviews (PRIOR) statement protocol [31]. This study selected and analyzed the studies according to the PRISMA (Preferred Reporting Items for Systematic Reviews and Meta-Analyses) guidelines. We used the Cochrane PICOS framework (population, intervention, comparison, outcomes, and study design (Table 1)). The methods used in all stages of screening, selection, and extraction, quality assessment, overlap management, analysis, and synthesis have been referenced so that our analysis can be replicated.

### 2.2. Inclusion and Exclusion Criteria

Systematic reviews were included in the review if they met the following selection criteria: (i) published in peer-reviewed journals; (ii) contain research questions on the acceptability and/or barriers to implementing digital interventions for people with dementia and/or caregivers; and (iii) participants of both sexes and aged 60 or over. The exclusion criteria were (i) articles that were not systematic reviews or meta-analyses; (ii) systematic reviews whose included primary studies were conducted exclusively during the COVID-19 pandemic; and (iii) robot interventions.

Selection of studies was performed independently by two independent reviewers (M.V.P and R.M.). Discrepancies were resolved through discussion with a third author (N.P.).

### 2.3. Data Extraction and Quality Assessment

Data from included studies was analyzed by two independent reviewers (M.V.P and R.M.) and extracted into Microsoft Excel^TM^ according to the points considered important to evaluate the acceptability factors and barriers to implementing digital interventions for people with dementia and/or their caregivers.

The methodological quality of all the included studies was assessed using the AMSTAR-2 checklist [35]. The AMSTAR-2 tool evaluates 16 domains, including seven critical items related to protocol registration, comprehensive literature searching, justification for study exclusions, risk of bias assessment, appropriateness of meta-analytical methods, consideration of risk of bias when interpreting results, and publication bias assessment. Following the AMSTAR-2 methodology [35] reviews were categorized as high quality (no or one non-critical weakness), moderate quality (more than one non-critical weakness), or low quality (one or more critical weaknesses). Quality assessments were performed independently by two reviewers (M.V.P. and R.M.), and any discrepancies were resolved through discussion with a third author (N.P.). The tool can categorize the quality of reviews according to seven critical areas and nine non-critical areas. The assessment was grouped into low, moderate, and high critical quality categories (Supplementary File S2).

To implement this umbrella review, we used the Preferred Reporting Items for Overviews of Reviews (PRIOR) statement protocol [31]. This study selected and analyzed the studies according to the PRISMA (Preferred Reporting Items for Systematic Reviews and Meta-Analyses) guidelines in Supplementary File S3. We followed the Consolidated Framework for Implementation Research (CFIR) to systematically organize and analyze the factors that influenced acceptance, barriers, and the impact of supportive technologies for individuals with mild cognitive impairment, dementia, and their caregivers (Table 2 and Table 3). The methods used in all stages of screening, selection, and extraction, quality assessment, overlap management, analysis, and synthesis have been referenced so that our analysis can be replicated.

To determine whether the same primary studies were included in more than one systematic review, we conducted a manual overlap analysis by cross-checking the reference lists of all five included reviews. Because of heterogeneous reporting formats and incomplete study identifiers in some reviews, a formal Corrected Covered Area (CCA) calculation was not feasible [36]. However, a qualitative comparison was performed to identify recurring studies and estimate the degree of overlap. Overlapping studies are documented and summarized in Supplementary File S2.

## 3. Results

### 3.1. Review Characteristics

**Figure 1:**
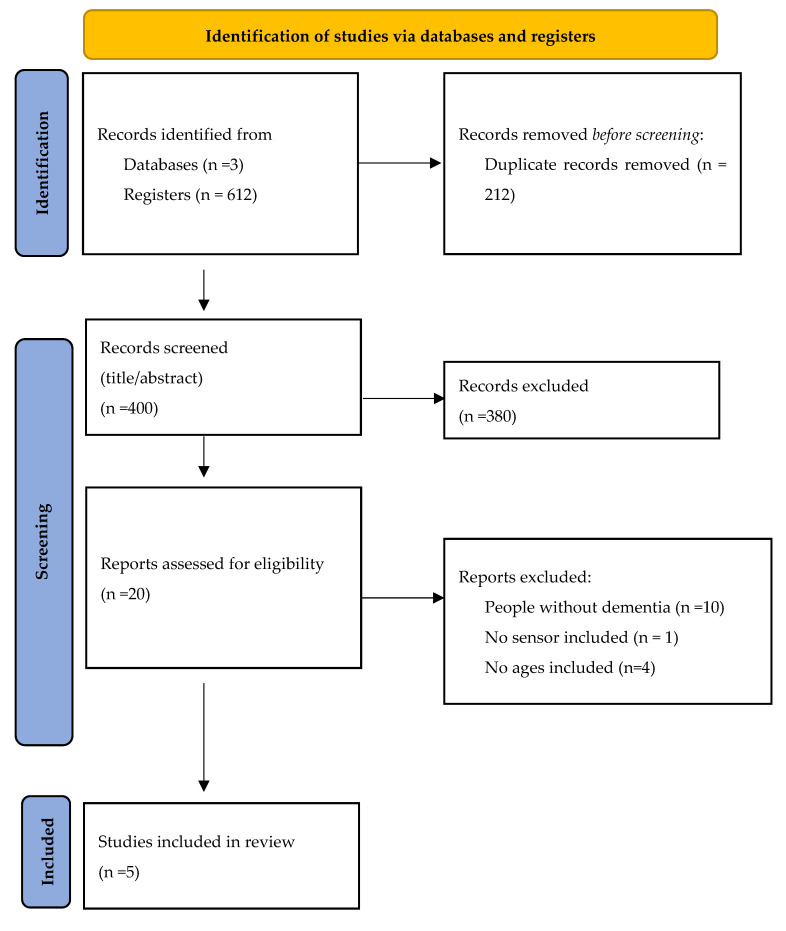
PRISMA (Preferred Reporting Items for Systematic Reviews and Meta-Analyses) diagram of the search and review process.

In the search, 612 studies in this area were identified from the three different databases. A total of 400 articles were identified after removing duplicates. After screening 400 records by title and abstract, 20 articles were retrieved for full-text assessment. Of these, 15 were excluded for the following reasons: populations without dementia (n = 10), no sensor or technology component (n = 1), and age criteria not met (n = 4). Ultimately, five systematic reviews met all inclusion criteria and were included in the umbrella review. The PRISMA flow diagram (Figure 1) reflects these numbers. Table 1 reports in detail each of the studies analyzed, thus presenting the study population, location of the intervention, time of the intervention, technology used and its category, assessment instruments, and location. Table 2 and Table 3 describe the intervention delivered, the reasons for acceptability and barriers, the impact of the intervention and limitations or future research on people with dementia and their carers, respectively. Figure 1 shows this process through a PRISMA diagram.

### 3.2. Overlap Assessment Results

Manual cross-checking of the primary studies across the five included reviews. A formal Corrected Covered Area (CCA) calculation [36] was approximated using confirmed duplicates only, yielding a CCA ≈ 3–4%, which indicates low overlap. Details of overlapping studies are presented in Supplementary File S2.

### 3.3. Study Characteristics

The systematic reviews included (Table 1) were published between 2019 and 2024 in three journals. Study designs included two systematic reviews [33,34], one meta-analysis [12], one record conducting both a systematic review and meta-analysis [15], and one systematic review and narrative synthesis [32]. The systematic reviews involved 93 studies. After checking the sample size of each study, we found that between 12 and 279 participants were included in the selected studies in a total of four articles. Caregivers ranged from 11 to 299 in a total of five articles.

The population included in this umbrella review were people with dementia and informal caregivers. The intervention was conducted at home [15,32,33,34], day and activity centers [32,33], nursing home [33], clinics [33], hospital [33], and an academic center [33]. Time of interventions ranged from 2 weeks to 12 months. The majority of articles used information and communication technology (ICT), but “Smart home” (e.g., ambient sensors for detecting falls, motion, or changes in daily routines), “Smart car” (e.g., in-vehicle alert systems and driver-assistance sensors to monitor cognitive performance and driving safety), “Wearables” (e.g., wristbands or GPS trackers to monitor mobility, heart rate, and location) and “Games technology” (e.g., cognitive training or exergaming platforms designed to support memory, attention, and engagement in people with dementia) were also used [33]. Assessment measures were divided into three categories: cognitive assessment of people with dementia, cognitive assessment of carers, and assessment of acceptability and barriers relating to the technology. The most used instruments to assess cognition were the Mini Mental State Examination (MMSE) and Zarit Burden Interview (ZBI). Acceptability and barriers were primarily assessed through a combination of quantitative questionnaires and qualitative methods. One study explicitly reported the use of the Unified Theory of Acceptance and Use of Technology (UTAUT) questionnaire [32], while others used semi-structured interviews, focus groups, and thematic analyses to explore user perceptions and experiences [12,15,33,34]. These approaches collectively examined dimensions such as perceived usefulness, ease of use, trust, and technological competence. Although standardized measurement tools were limited, qualitative methods provided complementary insights into the contextual and emotional factors influencing technology engagement.

Most studies were conducted in the USA, UK, and the Netherlands. Sweden, Denmark and South Korea are also mentioned less frequently.

### 3.4. People with Dementia

#### 3.4.1. Technology Intervention

Studies described medical follow-ups [33], ’check-up’ calls, information provision and communication [32], independence [32], activities of daily living [32,33], and cognition [32,33].

#### 3.4.2. Acceptance of Technologies

One systematic review reported on the acceptability factors of technologies in PWD. The acceptability factor mentioned had a positive impact on self-management [32]. This factor helps PWD to live more autonomously [32]. PWD become more capable for activities of daily living, mobility, and independence, therefore improving quality of life [32]. This type of technology also improves cognition and communication [32]. There is social engagement and social relationships [32].

#### 3.4.3. Barriers to Technologies

There are two systematic reviews in this umbrella that mention some barriers to the use of technologies by PWD [32,33]. Some barriers mentioned in these articles included difficulties in connecting, communicating, accessing, and using the technologies [32], need for technical expertise [32,33] severity of dementia [26], distrust and fear of being observed [32,33], forgetting to use and/or transport [32,33].

#### 3.4.4. Findings for People with Dementia

Technologies for people with dementia primarily support self-management, daily living activities, and cognitive stimulation. Acceptability was associated with perceived usefulness and autonomy enhancement, while barriers included difficulties with device operation, connectivity issues, distrust in being monitored, and disease severity. These findings are consistent with previous reviews [32,33].

### 3.5. Informal Caregivers

#### 3.5.1. Technology Intervention

Providing feedback on caregiving activities and monitoring the emotions of caregivers, in-person meetings and phone calls for monitoring and feedback [12,15,34]. There is more personal contact in telephone interventions compared to other types of carer interventions [12].

#### 3.5.2. Acceptance of Technologies

Two systematic reviews reported the acceptability factors of technologies for caregivers of PWD [15,34]. The acceptability factors mentioned were (1) they are more cost-effective than face-to-face interventions [15] and (2) the knowledge and skills needed to care for patients [15,34]. The use of mobile devices by carers can make it easier to call for help when needed, regardless of time or place [15]. Telephone interventions can be more cost-effective, being more accessible and requiring less technical knowledge than computer-assisted education technology [15]. Technology is a useful tool for providing resources and information and provides consistent contact with experts to help carers [15,34]. When provided with psycho-educational, multi-component, and psychotherapeutic interventions through digital technologies, there seems to be a relief from the emotional and physical burdens associated with caregiving, as well as improvements in depression and perceived social support [34].

#### 3.5.3. Barriers to Technologies

There are two systematic reviews that mention some barriers to the use of technology by PWD caregivers [12,15], including the need for technical knowledge [12,15]. Experience with technology decreases with age, which can make it difficult to implement [12].

### 3.6. Findings for Caregivers

For informal caregivers, digital interventions—such as web-based psychoeducation, mobile applications, and tele-support programs—were generally perceived as cost-effective and accessible alternatives to face-to-face interventions [12,15,34]. Acceptability was driven by perceived improvements in caregiving knowledge, confidence, and emotional support. However, barriers included limited digital literacy and anxiety about technology use, especially among older spousal caregivers.

### Acceptance and Transversal Barriers to Technology

Technologies are mainly used for monitoring, medical follow-up and/or feedback [12,15,32,33]. Telephone interventions are the most widely used and present the fewest barriers to use [12,15]. Technology seems to have a positive impact on self-management, being associated with a reduction in travelling and a reduction in the costs associated with face-to-face interventions, such as consultations or monitoring [12,15,32,33]. Self-management through technology may be associated with a reduction in carer burnout, reducing travelling between PWD [12,15]. The need for technical expertise seems to be a transversal barrier to technology [12,15,32,34]. This lack of knowledge can lead to difficulties with connection, communication, access, and utilization of technology [32].

### 3.7. Cross-Cutting Findings

Across both populations, technology facilitated remote monitoring, reduced travel burden, and improved access to health information. The most consistent barrier was the need for technical proficiency, which limited sustained engagement.

These cross-cutting findings emphasize the importance of training and simplified user design.

### Critical Appraisal

Four of the five studies were classified as high quality (Supplementary File S2). One study [32] was classified as low methodological quality. This study was considered at risk of bias (RoB) because it did not assess RoB in individual studies that were included in the review. Analyzing their reported classification of the risk of bias, we found that it ranged from low to high. One of the articles states that no article was included with a high risk of bias rating [12]. Another article states that it was unclear whether the studies made an effort to blind the participants or the evaluations of the results [15]. The bias analysis of the study [34] indicated a low risk of bias. Thus, there was some degree of risk of bias in the included studies. Finally, one review article included three articles with a high risk of bias, although the content of the included studies was of sufficient quality and robust enough to be included [32]. Therefore, we conclude that no significant discrepancies were found between the opinion of the original reviews and our own.

### 3.8. Effectiveness of Digital Interventions

Although the included systematic reviews reported heterogeneous outcome measures, consistent patterns emerged regarding intervention effectiveness. For people with dementia, technology-supported interventions demonstrated small-to-moderate improvements in cognitive engagement, communication, and daily functioning [32,33]. Among caregivers, digital psychoeducational and multicomponent programs were associated with reductions in depressive symptoms and improvements in self-efficacy and perceived social support [12,34]. However, one meta-analysis reported no significant effects on caregiver burden or stress [15], likely due to small sample sizes and the progressive nature of dementia. In contrast, another meta-analysis found small but statistically significant reductions in caregiver burden and depressive symptoms following technology-based interventions [12]. Overall, the evidence suggests that digital interventions are effective in enhancing psychosocial outcomes and caregiving competence, though their impact on objective health or burden measures remains inconclusive.

## 4. Discussion

This umbrella review summarized the acceptability factors and barriers reported by PWD and carers. In recent years, there has been an exponential increase in the development of research and recognition that these technologies can meet some of the needs of PWD and carers. Our umbrella review presents some acceptability factors and transversal barriers to the use of technology. Technology seems to have a positive impact on self-management for PWD by offering them tools and resources to manage their own health and well-being. It also has benefits in increasing access to health services and information for PWD and carers. In addition to access, technology can help reduce the costs associated with travelling for healthcare. Technology can be important for self-management, access to information, and reducing travel and costs. This type of technology can benefit, for example, people living in rural areas and/or those who have difficulty travelling, as well as reducing unnecessary trips to health services. However, the lack of technical knowledge seems to be the barrier most mentioned in the literature. A lack of technical knowledge about technology can compromise self-management and increase burden on carers.

One of the most consistent barriers identified across the included reviews was the lack of technological proficiency among both people with dementia and their caregivers. This challenge reflects broader issues of digital literacy, confidence, and accessibility in older populations. Several studies indicated that unfamiliarity with devices and software leads to frustration, reduced engagement, and abandonment of digital tools. For people with cognitive impairments, additional support and simplified interfaces are crucial to sustain participation. Similarly, caregivers, often older spouses, may experience anxiety and self-doubt when using technology. These findings highlight the need for structured training programs, continuous technical support, and age-appropriate design to overcome digital literacy gaps and enhance the long-term sustainability of digital interventions.

The use of technology can provide various advantages and disadvantages for both people with dementia and their caregivers. Technology has been used by PWD for medical monitoring, cognitive interventions and activities of daily living [32,33]. The type of intervention most used in this type of population was medical consultation. Technology for the caregiver has come to help provide feedback on care activities, monitor their emotions and provide support and education to caregivers [12,15,34]. The benefits include positive impacts on quality of life, behavioral problems, reduced burden on carers [12], reduced depression [12,34], and improving perception of social support [34]. However, in another article, the technology intervention had no significant effect on caregiver burden, depression or stress [15]. This inconsistency can be explained by the use of the ZBI questionnaire. The ZBI was developed to measure subjective burden [12,15]. The ZBI’s measurements consist of several aspects, and it is used to assess social and financial stressors, which are difficult to change with supportive interventions [12,15]. In addition, the type of technology can affect the results of studies [12,15]. The use of app-based interventions can increase the burden on carers [12,15]. It is also mentioned that carers dedicate their daily lives, time, energy, and money to PWD [15]. These factors can increase stress levels, leading to the development of depressive symptoms [15]. Another reason for these different outcomes may be that dementia is progressively degenerative and sometimes terminal [12]. Although technology is seen as useful, feelings of sadness, loss, and burden in carers often remain unchanged [12]. Another common reason for the lack of significance is insufficient statistical power resulting from small sample sizes [34]. Cost-effectiveness factors seem to be a determining factor in the acceptability of the technology by carers [15]. Remote services can offer practical advantages such as reduced travelling, associated costs [15], and access to care information [15,34]. This type of technology can improve quality of life for PWD and carers [15]. Improved quality of life can be associated with the quality of relationships between PWD and carers based on technology [15]. For the carer, it is a useful tool for acquiring the knowledge and skills needed to care for PWD [12,15,34]. Acquiring knowledge and skills through digital technologies may support caregivers and people with dementia in managing daily challenges, increase awareness and understanding of the disease, and potentially contribute to maintaining independence and delaying institutionalization [12,15,34]. The longer PWD remain at home with family care, the better their quality of life will be [15]. As such, technology includes factors to take into account such as increased self-management by PWD [32] and the knowledge and skills acquired by carers [12,15,34]. Therefore, technology is essential for improving quality of life, care, and later institutionalization. Despite these promising findings, it is important to acknowledge that not all studies demonstrated positive or significant effects of digital interventions. While one meta-analysis [15] found no statistically significant effects on caregiver burden, depression, or stress—likely due to small sample sizes and the progressive nature of dementia—another meta-analysis [12] reported small but significant reductions in caregiver burden and depressive symptoms following technology-based interventions. For people with dementia, evidence of clinical impact also remains limited: one review reported perceived gains in self-management and day-to-day functioning [32], whereas the monitoring-focused review did not demonstrate direct clinical effects [33]. This variability likely reflects methodological heterogeneity, including differences in intervention types, small sample sizes, short follow-up durations, and reliance on self-reported outcomes. Therefore, while digital technologies offer meaningful opportunities to enhance care and communication, their effectiveness should be interpreted with caution, as the evidence base remains preliminary and heterogeneous.

People with dementia and their caregivers find it difficult to use technology when they first receive it [32,33]. The appropriate use of technology requires an appropriate device and/or infrastructure and also digital literacy, which can often be related to sociodemographic factors, age, and severity of dementia [12,32,33]. Difficulty using technology and poor digital literacy are also reported in older people who do not have dementia [37,38]. Privacy can be another identified barrier to the use of technology by PWD, who report fear and suspicion of being observed [32,33]. Forgetting to use transport is also mentioned as a barrier [32,33]. With all these barriers, people with dementia often need support from their caregivers to access, install, and engage with technology. However, if carers provide support to make it easier for the person with dementia to access or engage with technology, this can lead to a decrease in the free time available, underloading their work. The participation and/or presence of the carer seems to be a requirement for satisfactory implementation [39]. We also need to understand that there are other barriers to the use of technology by caregivers. According to the caregivers’ sociodemographic data, the majority of caregivers were spouses and elderly people from rural areas [39]. Therefore, in an aging society with fewer younger carers, it is important to involve and support this important group, especially spousal carers. It is probable that sociodemographic factors and age are related to digital literacy, which can become a barrier to using this type of intervention [39,40].

To our knowledge, this is the first comprehensive study to synthesize evidence on acceptability factors and barriers to technology use by people with dementia and/or their carers. Although dementia is associated with old age [2], in some studies age is not referenced. Our umbrella review excluded some systematic reviews that referenced dementia at ages below 60. We therefore feel it necessary to draw attention to some conclusions from other articles that we consider pertinent. This type of intervention has the advantage of preserving the routine of the person with dementia [18], helping to limit mobility and the accessibility of care for populations in rural [41] and less economically developed contexts and/or populations from minority groups [39]. Technology-based intervention also seems to favor an improvement in the provision of care [39,41]. Caregivers feel more confident and qualified, improving caregiver–patient relationships [18,42]. These conclusions are in line with the results collected by our umbrella review.

However, there were some limitations that we think are pertinent. The use of technology by people with advanced dementia or sensory impairments may not be easy [18,41,42]. People with hearing and visual impairments are often excluded from studies [18,42] and several articles revealed limitations and concerns about the inclusion of this type of population [43,44]. We want to make people aware of the possibility of using subtitles for people with hearing impairments and headphones for people with visual impairments. It is also important to identify the degree of severity of dementia and to check the acceptability and barriers for the different stages of the disease.

## 5. Recommendations and Future Directions

This umbrella review included only five systematic reviews, which, although methodologically sound, may represent a relatively small body of evidence for each outcome evaluated. The heterogeneity of interventions, outcome measures, and populations across these reviews limits the comparability of findings and the extent to which conclusions can be generalized. Consequently, the results should be interpreted with caution, as they primarily provide an overview of existing trends and implementation challenges rather than definitive evidence of effectiveness. Based on the findings of this umbrella review, several recommendations can be made for research, practice, and policy. For research, we suggest conducting randomized controlled trials that include control groups and verifying the effects of technological interventions across different stages of dementia severity. Long-term studies with larger and more diverse samples are needed to assess sustainability and generalizability of outcomes. Special attention should be given to populations from less economically developed, rural, and minority backgrounds to better understand how interventions influence management, self-efficacy, preferences, and acceptability. Future studies should also explore cognitive assessment methods beyond the Mini Mental State Examination (MMSE), which may have limitations in capturing subtle cognitive changes. Additionally, research should address health inequalities, ethical concerns, data security, clinician and caregiver burden, and viable business models for large-scale implementation. It is also essential to develop standardized and validated qualitative assessment methods to evaluate the acceptability, usability, and experiential dimensions of digital health interventions in dementia care. Establishing common frameworks and metrics would facilitate comparison across studies and strengthen the evidence base for implementation.

For clinical practice, the results emphasize the need to incorporate digital literacy training for both caregivers and people with dementia into intervention protocols. Health professionals should be trained and supported in selecting accessible, user-friendly technologies, providing ongoing assistance, and minimizing frustration during technology adoption. The inclusion of technologies tailored for individuals with sensory impairments, such as visually impaired users, should also be prioritized to ensure inclusivity.

For policy and system-level implementation, strategies should promote equitable access to digital tools, subsidized devices, and programs that foster technological inclusion among aging populations. Collaboration between healthcare providers, technology developers, and policymakers can enhance usability, security, and scalability of digital health solutions, ensuring that technological innovation aligns with ethical standards and patient needs.

## 6. Conclusions

The systematic reviews and meta-analyses included in this umbrella review cover a diversity of designs and approaches. Populations and designs vary and much remains to be investigated and understood. Intervention through technology represents added value in solving the problem of accessibility in a way that is satisfactory for PWD, carers, and health professionals. In this sense, this type of technology is a potential solution to the current barriers imposed on access to conventional healthcare. Technology can be a tool for self-management, travel reduction, and cost reduction for PWD and their carers. Technology is also a useful tool for providing resources and the necessary information and provides consistent contact with specialists to help carers. However, the need for technical knowledge seems to be an obstacle which can lead to difficulties in connecting, communicating, accessing, and using technology. These difficulties seem to impose a burden of care. Our umbrella review aims to alert policy-makers and health services to the benefits and barriers to the use of technologies by PWD and carers. It seems imperative that future global community interventions in middle age-to-older adults address the literature regarding new technologies in order to try to mitigate this essential barrier, thus improving the potential effects that interactions with newer healthcare technologies may provide. Acceptability factors and barriers must also be taken into account in adoption and implementation to maximize the potential reach and effect on people with dementia and their families. Overall, digital health interventions show promise in supporting people with dementia and their caregivers by improving access, engagement, and psychosocial outcomes. However, given the limited number and heterogeneity of available systematic reviews, these findings should be interpreted cautiously. Future research using standardized methodologies and outcome measures is essential to strengthen the evidence base and guide large-scale implementation.

## Figures and Tables

**Table 1:** Characteristics of Included Studies.

Authors	Years	Sample, N	Population	Participant Characteristic	Location of Intervention	Intervention Time	Technology	Technology Category	Instrument	Location
Deeken et al. [12]	2018	29 studies	Informal Caregivers of PWD	Mean age 62 years. 11 to 299 Informal Caregivers	Home	30 days to 12 months.	Telephone, web-based interventions, DVD/video, or a combination of telephone and computer or DVD/video	ICT	CES-D, BDI, GDS, PHQ, BSI, ZBI, RMBPC, ICS, CSI, CAIVAS	NS
Lee et al. [32]	2021	11 Studies	PWD	59–92 years. 11 to 116 participants;	Home, Day and activity centers	Intervention sessions varied	Smartphones or tablets, computers, smartwatches, and followed by earpieces or headphones, app	ICT	MMSE The Unified Theory of Acceptance and Use of Technology Questionnaire	Denmark, Sweden, United Kingdom, Netherlands, United States
Piau et al. [33]	2019	26 studies	PWD	Mean age 64 to 89 years. 12 to 279 participants	Home, Adult day care center, nursing home, Remote clinic, Hospital, academic center	20 to 119 sessions 24 days to 9 months	Infrared motion sensors and magnetic contact door sensors; smart homes with combination motion and light sensors on the ceilings and combination door and temperature sensors on cabinets and doors; wrist-worn activity sensor device; GPS-enabled mobile phone; accelerometer; inertial sensors; IVRc technology; desktop computers; tablet; Nintendo Wii balance board; pill box	Smart home technologies and Smart car technologies, wearable ICT, game.	MMSE and CDR	NS
Shin et al. [15]	2022	5 Studies	Informal Caregivers of PWD	230 caregivers	Home	2 weeks to 3 months	Smartphone and mini-pad	ICT	CES-D, PSS, SSCQ, ZBI, QoL: AIOS, HADS, and saliva cortisol levels	USA, Netherlands, UK and South Korea
de-Moraes-Ribeiro et al. [34]	2024	22 Studies	Informal Caregivers of PWD	2761 Informal caregivers	Home	1 month to 2 years	Internet-based and mobile application	ICT	CES-D, ZBI, HADS, BDI, CCS, CSI, CGS, CSES, EQ5D-VAS, EQ5D+c, EuroQol, GHQ-12, GSE, HHI, ICECAP-O, IESS, MOS-SSS, MSPSS, NHP, NPI, PACS, PQOL, PSS, PSS-14, RCSS, RIS, RPFS, RSCSE, RSS, SF-12v2, SSCQ, STAI and WHOQOL-BREF	USA, Netherlands, France, UK, New Zealand, Canada, Germany, Spain, South Korea, Australia, India and Portugal

AIOS—Arizona Integrative Outcomes Scale; BDI—Beck Depression Inventory; BSI—Brief Symptom Inventory; CAIVAS—Caregiver Appraisal Inventory and Visual Analogue Scale; CCS—Caregiver Competence Scale; CDR—Clinical Dementia Rating; CES-D—Center for Epidemiological Studies Depression; CGS—Caregiver Grief Scale; CSES—Caregiver Self-Efficacy Scale; CSI—Caregiver Strain Instrument; EQ5D+c—European Quality of Life-5 Dimensions; EQ5D-VAS—EuroQol—Euro Quality of life—Visual Analog Scale; EuroQol—Euro Quality of life; GDS—Geriatric Depression Scale; GHQ-12—General Health Questionnaire-12; GSE—Generalized Self-Efficacy scale; HADS—Hospital Anxiety and Depression Scale; HHI—Herth Hope Index; ICECAP-O—Investigating Choice Experiments for the preferences of older people CAPability measure for Older people; ICS—Impact of Caregiving Scale; ICT—information and communication technology; IESS—Instrumental and Expressive Social Support scale; MMSE—Mini Mental State Examination; MOS-SSS—Medical Outcomes Study Social Support Survey; MSPSS—Multidimensional Scale of Perceived Support; NHP—Nottingham Health Profile; NPI—Neuropsychiatric Inventory; NS—not specified; PACS—Positive Aspects of Caregiving Survey; PHQ—Patient Health Questionnaire; PQOL—Perceived Quality of Life scale; PSS—Perceived Stress Scale; PSS-14—Perceived Stress Scale; PWD—people with dementia; RCSS—Revised Caregiving Satisfaction Scale; RIS—Eldercare Self-Efficacy Scale; RMBPC—Revised Memory and Behavior Problems Checklist; RPFS—Revised Piper Fatigue Scale; RSCSE—Revised Scale for Caregiving Self-Efficacy; RSS—Relative Stress Scale; SF-12v2—Short Form-12 item [version 2] health survey; SSCQ—Short Sense of Competence Questionnaire; STAI—State–Trait Anxiety Inventory; WHOQOL-BREF—World Health Organization Quality Of Life Brief version.

**Table 2:** Barriers and acceptance of technology for people with dementia.

Authors	Intervention	Acceptance	Barriers	Impact
Lee et al. [32]	Self-management concept, independence, activities of daily living, communication, and cognition	Positive impact on self-management	Difficulties in connecting, communicating, accessing, and using technology. Distrust and fear of being watched. Forgetting to use App.	Positive impact on the self-management concept
Piau et al. [33]	Real-life early detection and follow-up of cognitive function	NS	Distrust and fear of being watched. Forgetting to use and/or carry portable devices. Need for technical expertise. Dementia severity.	NS

NS—not specified; PWD—people with dementia.

**Table 3:** Barriers and acceptance of technology for caregivers of people with dementia.

Authors	Intervention	Acceptance	Barriers	Impact
Deeken et al. [12]	Treatments of behavioral activation, psychoeducation, coping strategies, supportive approaches, or cognitive behavioral therapy. Telephone-based cognitive behavioral therapy and used a telephone-based collaborative care management program with multiple modules, such as communication skills, stress management, and coping skills.	NS	Middle-aged and older adults have been shown to have lower self-efficacy and increased anxiety compared with younger adults	Positive impact on reducing depression and overload
Shin et al. [15]	Providing feedback on caregiving activities and monitoring emotions of caregivers with in-person meetings and phone calls for monitoring and feedback	More cost-effective than face-to-face interventions. The knowledge and skills needed to care for patients	Need for technical knowledge	Increased carer competence and quality of life. No significant effects on caregiver burden, depression or stress.
de-Moraes-Ribeiro et al. [34]	Psychoeducational, multicomponent and psychotherapeutic interventions	Psychoeducational interventions: The knowledge and skills needed to care for patients. Multicomponent interventions: Alleviates the emotional and physical burdens associated with caregiving. Psychotherapeutic interventions: improvements in depression and perceived social support.	NS	Psychotherapeutic interventions highlighted improvements in depression and perceived social support

NS—not specified.

## Data Availability

The datasets generated or analyzed during this study are available from the corresponding author upon reasonable request.

## Supplementary Materials

The following supporting information can be downloaded at https://www.mdpi.com/article/10.3390/jcm14227974/s1, File S1: Search strategy This Supplementary file provides the search strategy details, performed 28 September 2024; File S2: Quality assessment (AMSTAR 2) of included systematic reviews/meta-analysis. Overlap Matrix of Primary Studies Across Included Systematic Reviews. Technology Types Evaluated Across Included Studies; File S3: PRISMA 2020 Checklist [45].

## Abbreviations

PRISMA	Preferred Reporting Items for Systematic Reviews and Meta-analyses
PWD	People with Dementia
QoL	Quality of Life

## References

[1] Ganesan B., Gowda T., Al-Jumaily A., Fong K.N.K., Meena S.K., Tong R.K.Y. (2019). Ambient assisted living technologies for older adults with cognitive and physical impairments: A review. Eur. Rev. Med. Pharmacol. Sci..

[2] Gale S.A., Acar D., Daffner K.R. (2018). Dementia. Am. J. Med..

[3] Sekhon H., Sekhon K., Launay C., Afililo M., Innocente N., Vahia I., Rej S., Beauchet O. (2021). Telemedicine and the rural dementia population: A systematic review. Maturitas.

[4] Karlsson S., Bleijlevens M., Roe B., Saks K., Martin M.S., Stephan A., Suhonen R., Zabalegui A., Hallberg I.R., RightTimeCarePlace Consortium (2015). Dementia care in European countries, from the perspective of people with dementia and their caregivers. J. Adv. Nurs..

[5] Kim H., Jhoo J.H., Jang J.W. (2017). The effect of telemedicine on cognitive decline in patients with dementia. J. Telemed. Telecare.

[6] Kim S.K., Park M. (2017). Effectiveness of person-centered care on people with dementia: A systematic review and meta-analysis. Clin. Interv. Aging.

[7] Jackson D., Roberts G., Wu M.L., Ford R., Doyle C. (2016). A systematic review of the effect of telephone, internet or combined support for carers of people living with Alzheimer’s, vascular or mixed dementia in the community. Arch. Gerontol. Geriatr..

[8] Severs E., James T., Letrondo P., Løvland L., Marchant N.L., Mukadam N. (2023). Traumatic life events and risk for dementia: A systematic review and meta-analysis. BMC Geriatr..

[9] Farina N., Page T.E., Daley S., Brown A., Bowling A., Basset T., Livingston G., Knapp M., Murray J., Banerjee S. (2017). Factors associated with the quality of life of family carers of people with dementia: A systematic review. Alzheimer’s Dement. J. Alzheimer’s Assoc..

[10] World Health Organization (2021). Dementia. https://www.who.int/news-room/fact-sheets/detail/dementia.

[11] Dogra S., Dunstan D.W., Sugiyama T., Stathi A., Gardiner P.A., Owen N. (2022). Active Aging and Public Health: Evidence, Implications, and Opportunities. Annu. Rev. Public Health.

[12] Deeken F., Rezo A., Hinz M., Discher R., Rapp M.A. (2019). Evaluation of Technology-Based Interventions for Informal Caregivers of Patients With Dementia-A Meta-Analysis of Randomized Controlled Trials. Am. J. Geriatr. Psychiatry Off. J. Am. Assoc. Geriatr. Psychiatry.

[13] Leng M., Zhao Y., Xiao H., Li C., Wang Z. (2020). Internet-Based Supportive Interventions for Family Caregivers of People With Dementia: Systematic Review and Meta-Analysis. J. Med. Internet Res..

[14] Bayly M., Morgan D., Chow A.F., Kosteniuk J., Elliot V. (2020). Dementia-Related Education and Support Service Availability, Accessibility, and Use in Rural Areas: Barriers and Solutions. Can. J. Aging La Rev. Can. Du Vieil..

[15] Shin Y., Kim S.K., Kim Y., Go Y. (2022). Effects of App-Based Mobile Interventions for Dementia Family Caregivers: A Systematic Review and Meta-Analysis. Dement. Geriatr. Cogn. Disord..

[16] Covinsky K.E., Newcomer R., Fox P., Wood J., Sands L., Dane K., Yaffe K. (2003). Patient and caregiver characteristics associated with depression in caregivers of patients with dementia. J. Gen. Intern. Med..

[17] Wong P.K., Cheung G., Fung R., Koo S., Sit E., Pun S.H., Au A. (2008). Patient and Caregiver Characteristics Associated with Depression in Dementia Caregivers. J. Psychol. Chin. Soc..

[18] Caprioli T., Mason S., Tetlow H., Reilly S., Giebel C. (2023). Exploring the views and the use of information and communication technologies to access post-diagnostic support by people living with dementia and unpaid carers: A systematic review. Aging Ment. Health.

[19] Chiao C.Y., Wu H.S., Hsiao C.Y. (2015). Caregiver burden for informal caregivers of patients with dementia: A systematic review. Int. Nurs. Rev..

[20] Schulz R., Beach S.R. (1999). Caregiving as a risk factor for mortality: The Caregiver Health Effects Study. JAMA.

[21] Eisdorfer C., Czaja S.J., Loewenstein D.A., Rubert M.P., Argüelles S., Mitrani V.B., Szapocznik J. (2003). The effect of a family therapy and technology-based intervention on caregiver depression. Gerontol..

[22] Flandorfer P. (2012). Population ageing and socially assistive robots for elderly persons: The importance of sociodemographic factors for user acceptance. Int. J. Popul. Res..

[23] Blom M.M., Zarit S.H., Groot Zwaaftink R.B., Cuijpers P., Pot A.M. (2015). Effectiveness of an Internet intervention for family caregivers of people with dementia: Results of a randomized controlled trial. PLoS ONE.

[24] Czaja S.J., Charness N., Fisk A.D., Hertzog C., Nair S.N., Rogers W.A., Sharit J. (2006). Factors predicting the use of technology: Findings from the Center for Research and Education on Aging and Technology Enhancement (CREATE). Psychol. Aging.

[25] Davis F.D. (1989). Perceived usefulness, perceived ease of use, and user acceptance of information technology. MIS Q..

[26] Venkatesh V., Morris M.G., Davis G.B., Davis F.D. (2003). User acceptance of information technology: Toward a unified view. MIS Q..

[27] Chen K., Chan A.H.S. (2014). Gerontechnology acceptance by elderly Hong Kong Chinese: A senior technology acceptance model (STAM). Ergonomics.

[28] Peek S.T.M., Wouters E.J.M., van Hoof J., Luijkx K.G., Boeije H.R., Vrijhoef H.J.M. (2014). Factors influencing acceptance of technology for aging in place: A systematic review. Int. J. Med. Inform..

[29] Charness N., Boot W.R. (2009). Aging and information technology use: Potential and barriers. Curr. Dir. Psychol. Sci..

[30] Kuerbis A., Mulliken A., Muench F., Moore A.A., Gardner D. (2017). Older adults and mobile technology: Factors that enhance and inhibit utilization in the context of behavioral health. Ment. Health Addict. Res..

[31] Gates M., Gates A., Pieper D., Fernandes R.M., Tricco A.C., Moher D., Brennan S.E., Li T., Pollock M., Lunny C. (2022). Reporting guideline for overviews of reviews of healthcare interventions: Development of the PRIOR statement. BMJ (Clin. Res. Ed.).

[32] Lee A.R., Gerritzen E.V., McDermott O., Orrell M. (2021). Exploring the Role of Web-Based Interventions in the Self-management of Dementia: Systematic Review and Narrative Synthesis. J. Med. Internet Res..

[33] Piau A., Wild K., Mattek N., Kaye J. (2019). Current State of Digital Biomarker Technologies for Real-Life, Home-Based Monitoring of Cognitive Function for Mild Cognitive Impairment to Mild Alzheimer Disease and Implications for Clinical Care: Systematic Review. J. Med. Internet Res..

[34] de-Moraes-Ribeiro F.E., Moreno-Cámara S., da-Silva-Domingues H., Palomino-Moral P.Á., Del-Pino-Casado R. (2024). Effectiveness of Internet-Based or Mobile App Interventions for Family Caregivers of Older Adults with Dementia: A Systematic Review. Healthcare.

[35] Shea B.J., Reeves B.C., Wells G., Thuku M., Hamel C., Moran J., Moher D., Tugwell P., Welch V., Kristjansson E. (2017). AMSTAR 2: A critical appraisal tool for systematic reviews that include randomised or non-randomised studies of healthcare interventions, or both. BMJ (Clin. Res. Ed.).

[36] Pieper D., Antoine S.-L., Mathes T., Neugebauer E.A.M., Eikermann M. (2014). Systematic review finds overlapping reviews were not mentioned in every other overview. J. Clin. Epidemiol..

[37] Xie L., Zhang S., Xin M., Zhu M., Lu W., Mo P.K. (2022). Electronic health literacy and health-related outcomes among older adults: A systematic review. Prev. Med..

[38] Verma R., Saldanha C., Ellis U., Sattar S., Haase K.R. (2022). eHealth literacy among older adults living with cancer and their caregivers: A scoping review. J. Geriatr. Oncol..

[39] Lyu M., Zhao Q., Yang Y., Hao X., Qin Y., Li K. (2022). Benefits of and barriers to telehealth for the informal caregivers of elderly individuals in rural areas: A scoping review. Aust. J. Rural Health.

[40] Koivunen M., Saranto K. (2018). Nursing professionals’ experiences of the facilitators and barriers to the use of telehealth applications: A systematic review of qualitative studies. Scand. J. Caring Sci..

[41] Lazar A., Thompson H., Demiris G. (2014). A systematic review of the use of technology for reminiscence therapy. Health Educ. Behav. Off. Publ. Soc. Public Health Educ..

[42] Yi J.S., Pittman C.A., Price C.L., Nieman C.L., Oh E.S. (2021). Telemedicine and Dementia Care: A Systematic Review of Barriers and Facilitators. J. Am. Med. Dir. Assoc..

[43] Bittner A.K., Yoshinaga P.D., Rittiphairoj T., Li T. (2023). Telerehabilitation for people with low vision. Cochrane Database Syst. Rev..

[44] Simblett S., Greer B., Matcham F., Curtis H., Polhemus A., Ferrão J., Gamble P., Wykes T. (2018). Barriers to and Facilitators of Engagement With Remote Measurement Technology for Managing Health: Systematic Review and Content Analysis of Findings. J. Med. Internet Res..

[45] Page M.J., McKenzie J.E., Bossuyt P.M., Boutron I., Hoffmann T.C., Mulrow C.D., Shamseer L., Tetzlaff J.M., Akl E.A., Brennan S.E. (2021). The PRISMA 2020 statement: An updated guideline for reporting systematic reviews. BMJ.

