# Supplementary material for: Acceptance Factors and Barriers to the Implementation of Digital Interventions in Older People with Dementia and/or Their Caregivers: An Umbrella Review
Source: J Clin Med. 2025 Nov 10;14(22):7974. doi: 10.3390/jcm14227974 (PMC12653905; doi:10.3390/jcm14227974)
Supplement: Supplementary file 1 [file jcm-14-07974-s001.zip › jcm-3959677-supplementary/jcm-3959677-Supplementary file S1.pdf]

Supplementary file 1: Search strategy This Supplementary file provides the search strategy details, performed September 28th, 2024

Search strategy in PubMed September 28th, 2024

Set	Search terms	Result
1	(((((Elderly) OR ("Older population")) OR ("Older people")) OR ("Geriatrics"[Mesh])) OR ("Aged"[Mesh])) OR ("Aging"[Mesh])	6,4**29**,6**04**
2	((("Telemedicine"[Mesh]) OR ("Mobile Applications"[Mesh])) OR ("health informatics")) OR ("healthcare technology")	8**0.993**
3	((((implementation) OR (Barriers)) OR (Acceptance)) OR (Adherence)) OR (Restriction)	2,2**80**,5**10**
4	(((("Dementia"[Mesh]) OR ("Cognition Disorders"[Mesh])) OR ("mental deterioration*")) OR ("cognitive impairment*")) OR ("mild cognitive impairment*")	347,061
1+2+3+4	((((((((Elderly) OR ("Older population")) OR ("Older people")) OR ("Geriatrics"[Mesh])) OR ("Aged"[Mesh])) OR ("Aging"[Mesh])) AND (((("Telemedicine"[Mesh]) OR ("Mobile Applications"[Mesh])) OR ("health informatics")) OR ("healthcare technology"))) AND (((((implementation) OR (Barriers)) OR (Acceptance)) OR (Adherence)) OR (Restriction))) AND ((((("Dementia"[Mesh]) OR ("Cognition Disorders"[Mesh])) OR ("mental deterioration*")) OR ("cognitive impairment*")) OR ("mild cognitive impairment*"))	200

Search strategy in Scopus September 28th, 2024

Set	Search terms	Result
1	(((((Elderly) OR ("Older population")) OR ("Older people")) OR ("Geriatrics")) OR ("Aged")) OR ("Aging")	7,643,363
2	((("Telemedicine") OR ("Mobile Applications")) OR ("health informatics")) OR ("healthcare technology")	149,051
3	((((implementation) OR (Barriers)) OR (Acceptance)) OR (Adherence)) OR (Restriction)	4,150,438
4	("Dementia") OR ("Cognition Disorders") OR ("mental deterioration") OR ("cognitive impairment") OR ("mild cognitive impairment")	378,635
1+2+3+4	(Elderly) OR ("Older population") OR ("Older people") OR ("Geriatrics") OR ("Aged") OR ("Aging") AND ("Telemedicine") OR ("Mobile Applications") OR ("health informatics") OR ("healthcare technology") AND (implementation) OR (Barriers) OR (Acceptance) OR (Adherence) OR (Restriction) AND ("Dementia") OR ("Cognition Disorders") OR ("mental deterioration") OR ("cognitive impairment") OR ("mild cognitive impairment")	282

Search strategy in Web of Science September 28th, 2024

Set	Search terms	Result
1	(((((Elderly) OR ("Older population")) OR ("Older people")) OR ("Geriatrics")) OR ("Aged")) OR ("Aging")	1,881,718
2	((("Telemedicine") OR ("Mobile Applications")) OR ("health informatics")) OR ("healthcare technology")	94,012
3	((((implementation) OR (Barriers)) OR (Acceptance)) OR (Adherence)) OR (Restriction)	3,163,245
4	("Dementia") OR ("Cognition Disorders") OR ("mental deterioration") OR ("cognitive impairment") OR ("mild cognitive impairment")	330,111
1+2+3+4	((((((((Elderly) OR ("Older population")) OR ("Older people")) OR ("Geriatrics")) OR ("Aged")) OR ("Aging")) AND (((("Telemedicine") OR ("Mobile Applications")) OR ("health informatics")) OR ("healthcare technology"))) AND (((((implementation) OR (Barriers)) OR (Acceptance)) OR (Adherence)) OR (Restriction))) AND ((((("Dementia") OR ("Cognition Disorders")) OR ("mental deterioration")) OR ("cognitive impairment")) OR ("mild cognitive impairment"))	130
