# Supplementary material for: Acceptance Factors and Barriers to the Implementation of Digital Interventions in Older People with Dementia and/or Their Caregivers: An Umbrella Review
Source: J Clin Med. 2025 Nov 10;14(22):7974. doi: 10.3390/jcm14227974 (PMC12653905; doi:10.3390/jcm14227974)
Supplement: Supplementary file 1 [file jcm-14-07974-s001.zip › jcm-3959677-supplementary/jcm-3959677-Supplementary file S2.pdf]

(Supplementary file 2).

Quality assessment (AMSTAR 2) of included systematic reviews/ meta-analysis.

AMSTAR 2 criteria∗	Deeken et al.	Lee et al.	Piau et al	Shin et al.	de-Moraes-Ribeiro et al.
1. Did the research questions and inclusion criteria for the review include the components of PICO?	Yes	Yes	Yes	Yes	Yes
2. Did the report of the review contain an explicit statement that the review methods were established prior to conduct of the review and did the report justify any significant deviations from the protocol?	Yes	Yes	Yes	Yes	Yes
3. Did the review authors explain their selection of the study designs for inclusion in the review?	Yes	Yes	Yes	Yes	Yes
4. Did the review authors use a comprehensive literature search strategy?	Yes	Yes	Yes	Yes	Yes
5. Did the review authors perform study selection in duplicate?	Yes	Yes	Yes	Yes	Yes
6. Did the review authors perform data extraction in duplicate?	Yes	Yes	Yes	Yes	Yes

7. Did the review authors provide a list of excluded studies and justify the exclusions?	Yes	Yes	Yes	Yes	Yes
8. Did the review authors describe the included studies in adequate detail?	Yes	Yes	Yes	Yes	Yes
9. Did the review authors use a satisfactory technique for assessing the RoB in individual studies that were included in the review?	Yes	Yes	No	Yes	Yes
10. Did the review authors report on the sources of funding for the studies included in the review?	Yes	Yes	No	Yes	Yes
11. If meta-analysis was justified did the review authors use appropriate methods for statistical combination of results?	Yes	NMC	NMC	Yes	NMC
12. If meta-analysis was performed did the review authors assess the potential impact of RoB in individual studies on the results of the meta-analysis or other evidence synthesis?	Yes	NMC	NMC	Yes	NMC
13. Did the review authors account for RoB in individual studies when interpreting/	Yes	Yes	NMC	Yes	Yes

discussing the results of the review?					
14. Did the review authors provide a satisfactory explanation for, and discussion of, any heterogeneity observed in the results of the review?	Yes	Yes	Yes	Yes	Yes
15. If they performed quantitative synthesis did the review authors carry out an adequate investigation of publication bias (small study bias) and discuss its likely impact on the results of the review?	Yes	Yes	No	Yes	Yes
16. Did the review authors report any potential sources of conflict of interest, including any funding they received for conducting the review?	Yes	Yes	Yes	Yes	Yes

N=no, NMC=no meta-analysis conducted, PY=partial Yes, RoB=risk of bias, Y=yes.

Overlap Matrix of Primary Studies Across Included Systematic Reviews

Primary Study / Intervention	Deeken et al. (2019)	Shin et al. (2022)	Lee et al. (2021)	Piau et al. (2019)	de Moraes Ribeiro et al. (2024)
Beauchamp et al., 2005 – Telephone-based caregiver support	✓		✓		
Beauchamp et al., 2008 / 2010 – REACH II/III adaptations (telephone & web)	✓		✓		
Blom et al., 2015 / 2016 – *Mastery over Dementia* (web-based CBT)	✓	✓	✓		✓
Kajiyama et al., 2013 – Online caregiver support	✓		✓		
Kajiyama et al., 2018 – Mobile psychoeducation app		✓			✓
Chiu et al., 2009 – Web-based multi-component education	✓		✓		
Marziali & Donahue, 2006 – Telehealth videoconferencing	✓		✓		
Beauchamp et al., 2008 – Telephone stress management	✓				✓
Boots et al., 2016 – *Partner in Balance* (online self-management)			✓		✓
Dam et al., 2017 – Web-based caregiver support			✓		✓
Torkamani et al., 2014 – ICT monitoring system for caregivers	✓			✓	

Primary Study / Intervention	Deeken et al. (2019)	Shin et al. (2022)	Lee et al. (2021)	Piau et al. (2019)	de Moraes Ribeiro et al. (2024)
Lewis et al., 2010 – Online caregiver education	✓		✓		

Technology Types Evaluated Across Included Studies

Technology type	Examples	Authors
Web-based psychoeducation & support platforms	Structured websites, online CBT modules, discussion forums	12, 28, 30
Mobile applications (app-based programs)	Education apps, symptom trackers, reminders	12, 15, 30
Teleconsultation / tele-support	Video calls, telephone coaching, helplines	12, 28
Wearables	Wristbands, GPS locators, activity trackers	28, 29
Smart home / ambient sensors	Motion sensors, door sensors, bed mats, fall detectors	29
Digital biomarkers & home-based monitoring	Passive sensing, in-home activity signatures, digital cognitive markers	29
Game-based / cognitive training / exergames	Interactive cognitive tasks, Wii/Kinect, serious games	28, 30
GPS tracking / locator services	Standalone GPS tags, app-linked trackers	28, 29

Abbreviations: PWD = people with dementia; ADL = activities of daily living. Representative reviews reference numbers correspond to the manuscript numbering: 12 = Deeken et al. (2019), 15 = Shin et al. (2022), 28 = Lee et al. (2021), 29 = Piau et al. (2019), 30 = de Moraes Ribeiro et al. (2024).
